# High-Sensitivity C-Reactive Protein Levels and Metabolic Disorders in Obese and Overweight Children and Adolescents

**DOI:** 10.4274/Jcrpe.789

**Published:** 2013-03-21

**Authors:** Konstantinos Kitsios, Maria Papadopoulou, Konstantina Kosta, Nikolaos Kadoglou, Maria Papagianni, Kiriaki Tsiroukidou

**Affiliations:** 1 “G.Gennimatas” General Hospital, Department of Internal Medicine, Thessaloniki, Greece; 2 Aristotle University of Thessaloniki, Hippokration General Hospital, Third Department of Pediatrics, Thessaloniki, Greece; 3 Foundation of Biomedical Research, Academy of Athens, Athens, Greece

**Keywords:** High-sensitivity C-reactive protein, obesity, metabolic syndrome, impaired glucose tolerance, liver disease

## Abstract

**Objective:** To compare high-sensitivity C-reactive protein (hsCRP) levels in obese and overweight children and adolescents to normal-weight individuals as well as to compare hsCRP levels in overweight children/adolescents with and without additional metabolic disorders such as metabolic syndrome (MS), non-alcoholic fatty liver disease (NAFLD), and prediabetes.

**Methods:** 54 consecutive obese children and adolescents with a body mass index (BMI) ≥95th centile and 50 overweight children and adolescents with BMI values between 85th and 95th centiles were screened for MS, prediabetes and NAFLD. Serum hsCRP levels were measured in all the participants and in 40 age-matched normal-weight individuals (controls).

**Results:** HsCRP levels were significantly increased in obese and overweight subjects as compared to the control group, (0.61±1.08 vs. 0.05±0.18 mg/dL, p<0.001 and 0.33±0.25 vs. 0.05±0.18 mg/dL, p<0.001, respectively). HsCRP levels were similar between obese and overweight subjects (p=0.109). Obese and overweight children with NAFLD had significantly higher levels of hsCRP compared to their counterparts without NAFLD (0.78±1.4 vs. 0.34±0.31 mg/dL, p=0.016). The levels of hsCRP were comparable in the obese and overweight children/adolescents with and without MS and with or without prediabetes (0.95±1.66 vs. 0.35±0.27 mg/dL, p=0.096 and 0.43±0.34 vs. 0.53±1.0 mg/dL, p=0.589, respectively).

**Conclusions:** HsCRP is significantly elevated in children and adolescents with excess weight as compared to normal-weight individuals. In addition, children and adolescents with excessive weight and NAFLD show increased levels of hsCRP compared to their counterparts with normal liver.

**Conflict of interest:**None declared.

## INTRODUCTION

C-reactive protein (CRP) is a protein produced mainly from hepatocytes in acute episodes of inflammation or infection. In recent years, CRP has been established also as a marker of subclinical inflammation. Several studies have confirmed the role of subclinical inflammation in the course of atherosclerosis and have associated CRP levels with the metabolic syndrome (MS), type 2 diabetes mellitus (T2DM) and increased risk for cardiovascular disease (1,2). There are only a few published studies, with a small number of participants, on obese children and adolescents, which describe subclinical inflammation in these early ages, but these studies do not fully investigate the characteristics and clinical importance of subclinical inflammation (3).

The purpose of this study was to compare the levels of high-sensitivity CRP (hsCRP) in the serum of obese and overweight children and adolescents with those of normal-weight children. Furthermore, hsCRP levels of obese and overweight children with MS, prediabetes (impaired fasting glucose (IFG) and/or impaired glucose tolerance (IGT)) and non-alcoholic fatty liver disease (NAFLD) were compared with those of children and adolescents with increased body weight but without any other metabolic disorder.

## METHODS

One hundred and forty-four children and adolescents aged 6-17 years were included in the study. According to their body mass index (BMI) values, the participants were divided into 3 groups. Group A consisted of 54 obese children and adolescents (27 prepubertal) with BMI values ≥95th centile for their age and sex. In Group B, there were 50 overweight children and adolescents (19 prepubertal) with BMI values between the 85th and 95th centiles. In Group C, there were 40 children and adolescents (22 prepubertal) with BMI <85th centile (control group). The participants in groups A and B were children and adolescents who consecutively attended the pediatric outpatient obesity clinic of the 3rd Pediatric Department of the University of Thessaloniki at the Hippokratio General Hospital. All families were informed about the purpose of the study and written consent was obtained.

Weight was measured using high precision scales (accuracy 0.1 kg) and height was measured with a Harpenden stadiometer (accuracy of 1 mm). BMI was calculated according to the formula: weight (kg)/[height (m)2]. Greek population centiles, as published from the 1st Pediatric Department of the University of Athens, were used as reference values. Waist circumference (WC) was measured with a flexible tape (accuracy of 1cm) at the middle of the distance between the last rib and the iliac crest.

A liver ultrasound scan to diagnose NAFLD and an oral glucose tolerance test (OGTT) were performed in groups A and B. The participants were admitted to the unit in the morning, after a 12-hour fasting period. A cannula was inserted to the midcephalic and midcoronal vein. After the first sample (time 0), an oral solution of D-glucose (1.75 g/kg, max dose 75 g) was ingested within 2-3 minutes. All samples were transferred to the laboratory immediately for determination of glucose levels, which were calculated using the glucose hexokinase enzymatic method in the Architect 800c analyst (Abbott Laboratories, IL, USA, reference values: 75-100 mg/dL). American Diabetes Association (ADA) and International Diabetes Foundation (IDF) criteria were used to diagnose prediabetes (IFG: 100 mg/dL ≤ fasting glucose < 126 mg/dL and /or IGT: 140 mg/dL ≤ 2h glucose < 200 mg/dL).

MS was diagnosed according to the Cook criteria amended for the fasting glucose levels, where upper normal value was set to 100 mg/dL (4). WC values were plotted based on the centiles established by Fernandez et al (5) for USA children and adolescents of European origin, since there are no published reference data for the Greek population.

All liver ultrasound scans were performed and assessed by the same sonographer using a real-time 3.5 MHz transducer according to pre-defined standards (6).

Insulin resistance (IR) was calculated using the homeostasis model assessment for insulin resistance (HOMA-IR) formula: [fasting glucose (mmol/L)]?[fasting insulin (μU/mL)] /22.5. Insulin levels were calculated using the RIA method (reference values: 6-27 μU/mL). Hyperinsulinemia was defined as fasting insulin >20 μU/mL. ?sCRP levels were measured by a two-site chemiluminescent enzyme assay (reference values: 0.00-0.744 mg/dL).

**Statistical Analysis**

Quantitative variables were summed up in averages and standard deviations (SD). Student’s t-test was used to compare the averages in each group. The comparison of averages of all groups was done using the one-way ANOVA test. Tukey’s post-hoc analysis was used for qualitative comparisons. Normality of variables was tested with the Shapiro-Wilk test and no deviation was noted. Pearson test was used for the association of variables. SPSS edition 16.0 was used for the statistical analysis and the level of significance was defined as lower than 0.05 in both directions.

## RESULTS

The results of the between-groups comparisons for all the parameters studied (median±SD) are summarized in Tables 1, 2, 3.

HsCRP was significantly increased in obese and ?in overweight children compared to the control group (0.61±1.08 vs. 0.05±0.18 mg/dL, p<0.001 and 0.33±0.25 vs. 0.05±0.18 mg/dL, p<0.001, respectively), but the significance was not evident when the levels of obese children were compared to those of the overweight group (p=0.109). ?HsCRP was increased in prepubertal obese and in ?prepubertal overweight participants compared to prepubertal ?controls (0.48±0.39 mg/dL vs. 0.07±0.23 mg/dL, p<0.001 and 0.33±0.24 mg/dL vs. 0.07±0.23 mg/dL, p=0.001, respectively). HsCRP was also increased in both obese and overweight adolescents as compared to their normal-weight counterparts (0.74±1.5 mg/dL vs. 0.02±0.09 mg/dL, p=<0.001 and 0.33±0.27 vs. 0.02±0.09 mg/dL, p<0.001, respectively). However, hsCRP levels were comparable in prepubertal obese and overweight children (p=0.160) as well as in obese and overweight adolescents (p=0.182). Obese and overweight children with NAFLD had significantly higher levels of hsCRP compared to their counterparts without NAFLD (0.78±1.4 vs. 0.34±0.31 mg/dL, p=0.016). The levels of hsCRP did not differ between obese and overweight children with and without MS and with or without prediabetes (0.95±1.66 vs. 0.35±0.27 mg/dL, p=0.096 and 0.43±0.34 vs. 0.53±1.0 mg/dL, p=0.589, respectively).

Among obese youth, 22 prepubertal children and 22 adolescents (81.5%) had hsCRP levels >0.2 mg/dL. Among overweight individuals, 12 prepubertal children and 16 adolescents (56%) had hsCRP>0.2 mg/dL, while in the control group, hsCRP levels exceeded 0.2 mg/dL in 4 prepubertal children and in 1 adolescent (12.5%).

MS was diagnosed in 17 (31.5%) of the obese and in 4 (8%) of the overweight children and adolescents. Among obese individuals with MS, 94% had high-density lipoprotein cholesterol (HDL)-cholesterol levels <40 mg/dL and 64.7% had triglyceride levels >110 mg/dL; 58.8% of these patients had fasting plasma glucose (FPG) levels >100 mg/dL and 23.5% had blood pressure values >90th percentile. Similarly, 75% of the overweight participants had low HDL and elevated triglyceride levels, and in 50%, FPG levels were elevated. Prediabetes was found in 24 (44.4%) of the obese and in 12 (24%) of the overweight children and adolescents. Among the obese participants with prediabetes, 16 (29.6%) had IFG, 3 (5.6%) had IGT, and 5 (9.3%) had both IFG and IGT. Among the overweight children and adolescents with prediabetes, 8 (16%) had IFG, 3 (6%) had IGT, and only 1 (2%) had both IFG and IGT. NAFLD was diagnosed in 24 (44.4%) of the obese and in 9 (18%) of the overweight children and adolescents. Among all the participants, only 4 obese adolescents had blood pressure values that exceeded the 90th percentile for their age, sex, and height. Acanthosis nigricans was diagnosed in 4 prepubertal and 8 pubertal obese participants and in 2 prepubertal and 5 pubertal overweight individuals. The results of the comparison between children and adolescents with and without MS, with and without prediabetes, with and without NAFLD are presented in Tables 4, 5, and 6, respectively.

Among all the parameters studied, Pearson’s correlation test showed a positive correlation only between hsCRP levels and IR both in obese (r=0.275, p=0.05) and overweight (r=0.513, p=0.001) children and adolescents.

## DISCUSSION

CRP is an acute-phase protein, produced mainly from hepatocytes shortly after an inflammatory stimulus. The production of CRP is regulated by several cytokines including IL-1, IL-6, and TNFα which are secreted locally in the area of harmed tissue. The gene responsible for CRP synthesis is located in chromosome 1 (1q21-q23) and its function is regulated at the transcription level (7). High levels of CRP are strongly associated in epidemiological studies with obesity, cardiovascular disease, MS, IR, increased FPG, and T2DM (8,9,10). Nevertheless, there is still not enough evidence to establish a clear etiological relation between CRP and atheromatosis although there is a strong correlation between CRP as a marker of systemic inflammation and the evolution of atheromatosis. Different CRP gene polymorphisms expressed as different quantitative phenotypes of the molecule in blood circulation are not associated with cardiovascular disease, hypertension and IR (11,12).

In this study, hsCRP levels were significantly higher in the obese and overweight groups compared to the control group, and there was a positive correlation with IR in both groups. These results are in accordance with data previously published. Shin et al (13) reported increased hsCRP levels in obese compared to non-obese children. Similarly, Giannini et al (14) compared the levels of inflammatory markers between obese and normal-weight prepubertal children and reported significantly higher levels of hsCRP in the obese participants. In a small cross-sectional study, Akinci et al (15) reported elevated hsCRP levels in overweight compared to normal-weight children. In a recent publication from Argentina (16), obese and overweight adolescents presented with higher levels of hsCRP in comparison to non-obese, and hsCRP levels positively correlated with WC measurements and BMI. A large epidemiological study including 2300 children aged 9-15 years in Norway also showed a strong correlation of hsCRP with WC (17). Semiz at al (18) have reported a correlation between hsCRP levels and cardiovascular risk in obese children and adolescents.

In our study, we found no difference in hsCRP levels between obese and overweight individuals, suggesting that hsCRP is increased in the early stages of accumulation of weight towards obesity. We are not aware of any publications regarding a comparison of hsCRP levels between obese and overweight children and adolescents.

In our study, obese and overweight children with MS had similar hsCRP levels to their counterparts without MS. Similarly, Cizmecioglu et al (19) reported no significant difference in hsCRP levels between overweight and obese children and adolescents with and without MS. On the contrary, in a study from Spain (20), obese children with MS presented higher levels of hsCRP compared to those without the syndrome. Among more than 400 Brazilian children and adolescents participating in an epidemiologic study (21), those with MS had significantly higher levels of hsCRP compared to those without MS, but the latter group included also normal-weight individuals. Furthermore, in this study, hsCRP was found to correlate significantly with IR, WC and most of the other components of MS. Ford et al (22), in a large study of 1400 adolescents aged 12-17 years, also reported higher levels of hsCRP in individuals who had MS.

We did not find any difference in hsCRP levels between obese and overweight children with and without prediabetes. Although evidence from direct comparisons seems to be lacking, it is noteworthy that in the above-mentioned Brazilian study (21), the only MS component not related to CRP was fasting glucose level. It is possible that the increase in hsCRP is a pretty early consequence of excessive weight, preceding the manifestation of any impairment in glucose metabolism.

Free fatty acid accumulation in the liver results in increased production of hsCRP and oxidative stress by activating the B kappa pathway (NFκB) in hepatocytes and Kupffer cells. Increased visceral fat has the same effect and all these factors play an important role in the pathogenesis of NAFLD and non-alcoholic steatohepatitis (NASH) by interfering in the process of hepatocytic apoptosis and periportal fibrosis (23). In our study, we showed that obese and overweight children with NAFLD had significantly higher hsCRP levels compared to their counterparts without NAFLD. In accordance with our findings, Weghuber et al (24) also reported significantly elevated hsCRP levels in obese children with NAFLD compared to obese children with normal hepatocellular lipid content. In contrast, in a prospective cohort study on overweight and obese children in Israel (25), no significant association was found between CRP and NAFLD. Sartorio et al (26) explored the predictors of NAFLD in obese children and reported that CRP was not an independent predictor of the disease. Recently, in a study in obese adults (27), based on liver biopsies, hsCRP level was found to be a potential marker of steatosis but not of the severity of NAFLD.

It is noteworthy that 80% of the obese children and more than 50% of the overweight participants in our study had hsCRP levels >0.2 mg/dL. In a recent trial of primary prevention of cardiovascular disease in 18 000 healthy adults with normal lipid profile but hsCRP levels >0.2 mg/dL, treatment with a statin for two years resulted in 37% reduction of hsCRP and in significant reduction of acute coronary events, ischemic strokes and deaths from cardiovascular disease (28). Since most of the participants in our study would be eligible for such a prevention strategy, routine measurement of hsCRP levels in children and adolescents with excessive weight might prove to be a useful tool in planning clinical interventions.

There are certain limitations in the interpretation of the results of our study. IR was assessed by using HOMA-IR, a well-validated index in adults, but still not extensively standardized in children and adolescents. Hyperinsulinemic euglycemic clamp is the gold standard in the assessment of IR, but this method is difficult to apply in children. In the Cook criteria for the diagnosis of MS, IR is not prerequisite. In our study, dyslipidemia was the main component of the MS both in the obese and overweight participants. HsCRP was positively correlated with IR but not with lipid disorders and this finding may be due to the absence of a difference in hsCRP levels between children and adolescents with and without MS. Finally, our study was underpowered to detect possible differences in pubertal participants across Tanner stages, since 85% of them were in stages 4 and 5 at the time of their evaluation.

In conclusion, obese and overweight children and adolescents in our study had significantly elevated hsCRP levels compared to their normal-weight counterparts. However, there was no difference in the levels of this inflammation marker between obese and overweight children and adolescents or between obese and overweight children and adolescents with and without MS as well as between those with and without prediabetes. Finally, we did show that children and adolescents with excessive weight and NAFLD had significantly higher levels of hsCRP compared to their counterparts with normal liver. Given the controversies encountered in the literature on this issue, more studies are needed to further elucidate the relationship of CRP with various metabolic disorders in childhood and adolescence.

## Figures and Tables

**Table 1:**
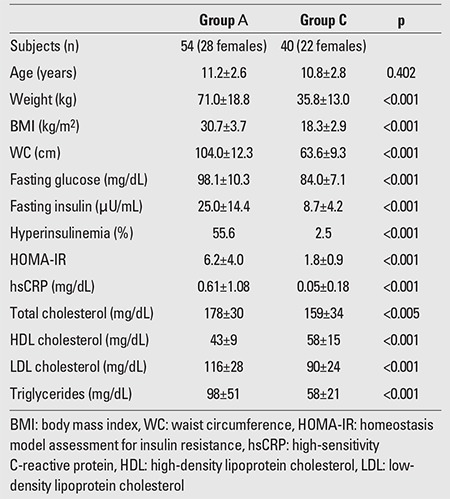
Comparison of obese children and adolescents (Group A) with the controls (Group C)

**Table 2:**
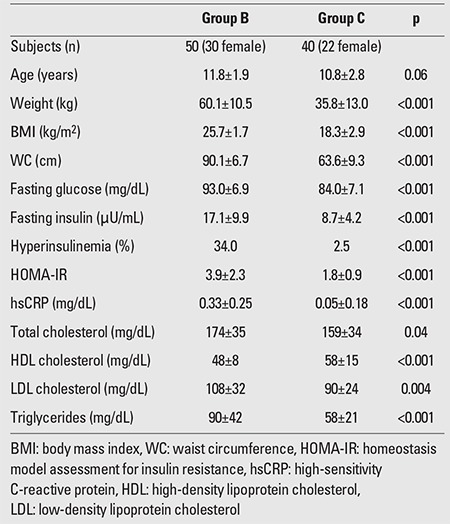
Comparison of overweight children and adolescents (Group B) with the controls (Group C)

**Table 3:**
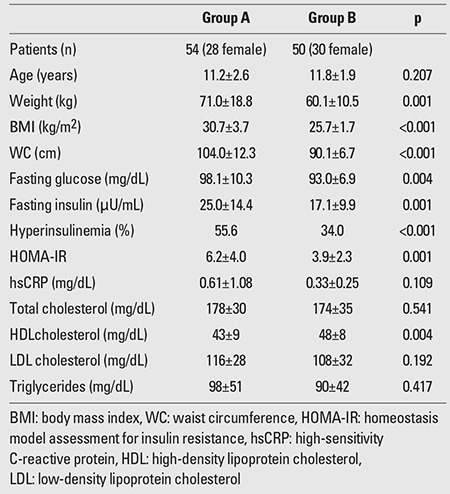
Comparison of obese (Group A) and overweight (Group B) children and adolescents

**Table 4:**
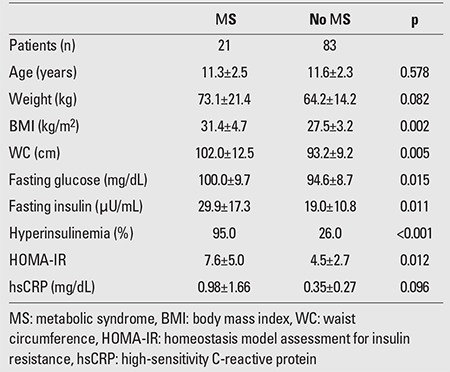
Comparison of obese and overweight children and adolescents with and without MS

**Table 5:**
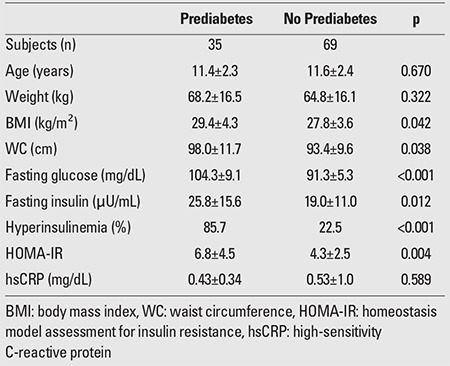
Comparison of obese and overweight individuals with and without prediabetes

**Table 6:**
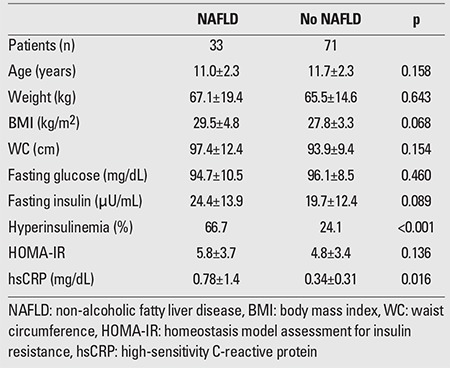
Comparison between obese and overweight children and adolescents with and without NAFLD
